# Mitochondrial DNA reveals high maternal diversity within a weak breed structure in native Kazakhstani horses

**DOI:** 10.3389/fgene.2026.1874969

**Published:** 2026-07-06

**Authors:** Daniya Ualiyeva, Kairat Dossybayev, Tilek Kapassuly, Altynay Kozhakhmet, Zhassulan Kozhanov, Kirill Kryukov, Masanori Arita, Merey Torekhanov, Aibyn Torekhanov

**Affiliations:** 1 Kazakh Research Institute of Livestock and Fodder Production, Almaty, Kazakhstan; 2 Institute of Genetics and Physiology, MSHE of the Republic of Kazakhstan, Almaty, Kazakhstan; 3 Faculty of Biology and Biotechnology, Farabi University, Almaty, Kazakhstan; 4 Bioinformation and DDBJ Center, National Institute of Genetics, Mishima, Shizuoka, Japan; 5 Center for Genome Informatics, Joint Support-Center for Data Science Research, Research Organization of Information and Systems, Mishima, Shizuoka, Japan; 6 Department of Informatics, National Institute of Genetics, Mishima, Shizuoka, Japan

**Keywords:** domestic horse, evolution, Kazakhstan, mitochondrial DNA, population genetics

## Abstract

Understanding the genetic diversity and evolutionary history of domestic horses (*Equus caballus*) is essential for reconstructing their population dynamics and origins. In this study, we analyzed mitochondrial DNA variation (COI and cytb) in six Kazakhstani horse populations representing four native breeds (Kazakh, Kostanay, Adai, and Mugalzhar) to assess genetic diversity, population structure, and evolutionary relationships within a broader phylogenetic framework. High haplotype diversity combined with low nucleotide diversity revealed population expansion and admixture. Population structure analyses revealed weak genetic differentiation and a lack of breed-specific structuring, with most variation occurring within populations. Phylogenetic reconstruction and haplotype network analyses showed that Kazakhstani horses are interspersed among global domestic lineages, reflecting extensive historical connectivity and admixture. Demographic analyses based on neutrality tests and mismatch distributions support signals of ancient population expansion, while the multimodal distribution patterns suggest a complex demographic history involving population substructure and multiple expansion events rather than a single, sudden expansion. Times since expansion were calculated to be around 205 Kya. Divergence time estimates place the diversification of all caballine horses, including Kazakhstani horses, within the Pleistocene (0.89 Mya) with the majority of them grouping with different horse breeds across the world. These results indicate that the maternal genetic structure of Kazakhstani horses has been shaped by a combination of ancient evolutionary processes and more recent demographic dynamics, including recurrent gene flow across Eurasian steppe environments. Overall, this study highlights a reticulate evolutionary history of domestic horses, emphasizing the role of long-term connectivity and population expansion in shaping mitochondrial diversity.

## 1 Introduction

The family Equidae includes modern horses, zebras, and donkeys (MacFadden, 1986; Wilson and Reeder, 2005). Among domesticated animals, the horse occupies a unique position in human history, playing a central role in transportation, warfare, agriculture, and cultural development across Eurasia. Its remarkable ecological adaptability has enabled its use across diverse environments and functions, ranging from subsistence production to sport and therapeutic applications.

Archaeological and genetic evidence indicates that the Eurasian steppe played a major role in early human–horse interactions and the development of horse management practices, with strong evidence from territories corresponding to present-day Kazakhstan. The Botai settlement, dated to approximately 5,500 years before present (Levine, 1999; Olsen Zeder et al., 2006), provides some of the earliest evidence of intensive horse exploitation, including milking, harnessing, and corralling. Although recent genomic studies suggest that Botai horses were not direct ancestors of most modern domestic horse lineages, the site remains fundamental for understanding early equine management in the Eurasian steppes. Additional contemporaneous sites, such as Borly4, further support the importance of this region in the emergence of horse husbandry practices (Outram et al., 2009).

Since antiquity, horses have been integral to the subsistence and cultural traditions of Central Asian nomadic societies. In Kazakhstan, horse breeding remains a major component of national heritage, supporting meat and dairy production, transport, and equestrian sport. Extensive natural pasture systems and harsh continental climates have favored the development of hardy local breeds adapted to year-round grazing and long-distance mobility.

Several indigenous horse breeds have been established through regional selection and breeding programs, including the Kushum, Kostanay, Adai, and Mugalzhar breeds. The Kushum and Mugalzhar breeds are dual-purpose horses used for both meat and milk production, whereas the Kostanay and Adai breeds are classified as riding horses. The Kazakh horse comprises two intrabreed types: Jabe (Zhabe) and Naiman. Adai horse breed was recently elevated to the rank of breed (Sizonov et al., 2023), previously (Nechaev et al., 2005; Rzabaev and Rzabaev, 2016) being considered as an intrabreed type of the Kazakh horse breed that underwent historical crossbreeding with riding horses from neighboring regions (Asanbaev, 2016; Assanbayev et al., 2019). The Kostanay horse breed was developed as a riding and harness type, with its formation beginning in the late 19th century in the Kostanay region. The breeding program involved crossing Kazakh local mares with stallions of the Kalmyk, Strelets, and Don breeds, followed by further improvement of the resulting crossbred offspring using Thoroughbred stallions. The breed was officially recognized and approved through state evaluation in 1951. The Mugalzhar breed is only a milk-meat-oriented breed, established by reproductive crossing of local horse populations with selected Jabe stallions, resulting in animals well adapted to year-round pasture conditions and climatic extremes (Barmintsev, 1958; Rzabayev, 2007).

Over the last 2 decades, molecular genetic research on indigenous horse populations of Kazakhstan has progressed from classical marker systems to genome-scale analyses. Early investigations based on mitochondrial DNA sequences revealed substantial maternal diversity and complex phylogeographic structure, reflecting multiple origins and extensive historical gene flow among steppe horse populations (Cieslak et al., 2010; Achilli et al., 2012). *Cytb*-based analyses of Kazakh horses demonstrated high haplotype diversity and the presence of multiple maternal lineages, indicating a heterogeneous mitochondrial gene pool shaped by long-term admixture (Gemingguli et al., 2016). Microsatellite (STR) studies further confirmed high genetic variability and relatively weak differentiation among regional populations, consistent with extensive gene flow and traditional herd management practices (Orazymbetova et al., 2023; Seleuova et al., 2018). In contrast to the high maternal diversity, studies of paternal inheritance revealed extremely limited Y-chromosome variation, suggesting strong historical bottlenecks in male lineages associated with breeding practices typical of domestic horses (Nguyen et al., 2020).

More recent studies have employed high-resolution genomic approaches. Genome-wide SNP analyses across indigenous breeds demonstrated considerable individual genetic variation but relatively limited differentiation between breeds, indicating that Kazakh horses largely represent a heterogeneous landrace shaped by long-term environmental adaptation and pastoral breeding systems (Pozharskiy et al., 2023). Advances in long-read sequencing technology have further enabled the generation of high-quality *de novo* genome assemblies for the Jabe intrabreed type, providing important reference resources for comparative genomics, functional studies, and conservation genetics (Assanbayev et al., 2024). Despite these advances, most of the previous investigations have focused on individual breeds, localized populations, or specific genetic systems rather than providing a comprehensive evaluation of maternal lineage relationships across multiple indigenous horse breeds. Consequently, phylogenetic relationships, divergence history, and population structure of regional maternal lineages remain incompletely resolved, particularly based on complete mitochondrial protein-coding gene sequences.

Mitochondrial protein-coding genes provide a powerful framework for reconstructing maternal evolutionary history and population structure. In the present study, the *cytochrome c oxidase subunit I* (*COI*) and *cytochrome b* (*cytb*) genes were selected because they combine a relatively conserved sequence architecture with sufficient phylogenetically informative variation for evaluating interbreed relationships and maternal lineage differentiation (Kelle et al., 2014; Galtier et al., 2009; Hebert et al., 2003; Irwin et al., 1991). Compared with the mitochondrial *D-loop* region, which evolves rapidly and is highly informative for recent demographic events, protein-coding genes provide a more stable phylogenetic signal and lower levels of homoplasy, making them suitable for comparative analyses across geographically diverse horse populations. In addition, complete mitochondrial genome sequencing was beyond the scope of the present study due to sample and resource limitations. Therefore, analysis of complete *COI* and *cytb* sequences was used as a targeted approach to assess maternal genetic diversity, phylogenetic relationships, and evolutionary structure among indigenous horse breeds of Kazakhstan.

In this study, we investigate the genetic diversity and evolutionary relationships of indigenous horse populations from Kazakhstan using complete sequences of the mitochondrial *COI* and *cytb* genes. Specifically, we aim to (i) reconstruct phylogenetic relationships between Kazakh horse lineages and other domesticated horses, (ii) estimate divergence times of Kazakhstani *Equus caballus* from the most recent common ancestor (MRCA), and (iii) characterize the genetic structure of local populations. By integrating mitochondrial sequence data with comparative analyses across domesticated lineages, this study provides new insights into the evolutionary history of regional equine populations and contributes to the genetic characterization and conservation of indigenous horse breeds.

## 2 Materials and methods

### 2.1 Sample collection

**FIGURE 1:**
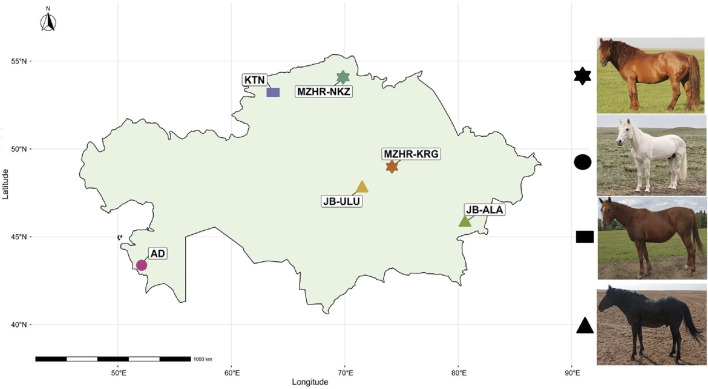
Sampling localities of the Kazakhstani horse breeds of *Equus caballus*. AD - Adai breed; JB-ULU - Jabe type of Kazakh horse from Ulytau region; JB-ALA - Jabe type of Kazakh horse from Almaty region; KTN - Kostanay breed; MZHR-KRG - Mugalzhar breed from Karaganda region; MZHR-NKZ - Mugalzhar breed from North Kazakhstan region.

All experimental procedures were approved by the Local Bioethics Committee of the LLP « Kazakh Research Institute of Animal Husbandry and Fodder Production» (Conclusion of the Bioethics Committee №11 from 5 April 2022, Almaty, Kazakhstan). Hair follicle samples were collected from individuals with proper documentation of their pedigree information. Samples were taken by a vet and stored dry in biobank of the Institute of Livestock and Fodder Production. No animals were sedated specifically for this study. Totally, 60 samples were collected from 3 different breeds and one Kazakh horse of Jabe type across Kazakhstan (Figure 1) comprising: Mugalzhar breed (MZHR-NKZ): population 1 (n = 10, North Kazakhstan region, “Karatomar” farm); (MZHR-KRG) population 2 (n = 10, Karaganda region, “Bakytbek” farm); Kostanay breed (KTN): population 3 (n = 10, Kostanay region, “Kazak tulpary” farm); Adai breed (AD): population 4 - (n = 10, Mangystau region, “Kuanysh” farm); Jabe type: population 5 - Jabe type 1 (JB-ALA), (n = 10, Almaty region, “Akimbekov” farm); population 6 is Jabe type 2 (JB-ULU) (n = 10, Ulytau region, “Orazaly” farm). MtDNA sequences generated in this study were deposited in GenBank under accession numbers (PZ226764-PZ226823; PZ232657-PZ232716) (Supplementary Table S1).

### 2.2 Laboratory protocol

Genomic DNA was isolated from a hair follicle using the standard proteinase K digestion and phenol-chloroform extraction procedures (Sambrook et al., 1989), after washing with excess NTE (0.05 M Tris-HCl, 0.01 M NaCl, 0.02 M EDTA, pH 9.0) to remove possible proteinase or PCR inhibitors. Polymerase chain reaction (PCR) was used to amplify the complete sequences of *COI* (1545 bp) and *cytb* (1140 bp) protein-coding genes. The *COI* region was amplified using the universal primer pair *COI*-long (f) (5934) (5-AAC​CAC​AAA​GAC​ATT​GGC​AC-3) and *COI*-long (r) (6597) (5-AAGAATCAGAATARGTGTTG-3). *Cytb* was amplified using the primer pair *cytb* (f) (15150) (5-GAGGMCAAATATCATTCTGAGG-3) and *cytb* (r) (15607) (5-TAGGGCVAGGACTCCTCCTAGT-3) (Townzen et al., 2008). Amplification was performed in a total volume of 50 µL containing 50 mM KCl, 10 mM Tris-HCl, 1.5 mM Mg2+, 200 µmol of each dNTP, 0.2 µmol of each primer, 2 U of Thermofisher Taq DNA Polymerase (Thermofisher, USA) and approximately 10 ng of genomic DNA. Thermal cycling conditions used to amplify the genes were: pre-denaturation at 94 °C for 4 min, followed by 35 cycles of denaturation at 94 °C for 30 s, annealing at 58 °C–60 °C (54 °C–60 °C for *COI*) for 30 s, and extension at 72 °C for 60 s (*COI*) and 90 s (*cytb*), respectively; and a final extension of 10 min at 72 °C; then stored at 4 °C. PCR product sizes were visualized by electrophoresis on a 1% agarose gel and then purified using a Qiagen PCR purification kit and subsequently sequenced for both strands using an ABI 3730 automated sequencer (Applied Biosystems, Inc.) according to the manufacturer’s instructions.

Most of the equine phylogeographic studies have focused on the hypervariable mitochondrial *D-loop* region. In contrast, the present study examined complete mitochondrial protein-coding genes (*COI* and *cytb*) to provide complementary phylogenetic information based on more conserved maternal markers.

### 2.3 Data analysis

#### 2.3.1 Sequence diversity

Sequences were aligned and translated into amino acid sequences using MEGA v. X (Kumar et al., 2018) to verify the data. *COI* and *cytb* two gene segments were concatenated using MEGA v. X with the default options. Unique haplotypes were determined using DnaSP v6 (Rozas et al., 2017). Basic sequence statistics and molecular indices, such as the number of haplotypes, polymorphic sites, haplotype diversities (h) and nucleotide diversities (π) were quantified with ARLEQUIN v3.5 (Excoffier and Lischer, 2010) (Table 1). Uncorrected *p*-distances were calculated using MEGA v. X (Kumar et al., 2018) (Table 2).

**TABLE 1:** Molecular indices of populations of Kazakhstani *Equus caballus* inferred from concatenated COI and cytb gene sequences.

Population	N	Nh	*S*	*m*	*k*	*Hd* ± st.d	π ± st.d
MZHR-NKZ	10	5	21	21	5.000	0.667 ± 0.163	0.00186 ± 0.00060
MZHR-KRG	10	8	27	27	10.111	0.956 ± 0.059	0.00377 ± 0.00033
KTN	10	3	15	15	5.066	0.511 ± 0.164	0.00189 ± 0.00062
AD	10	7	26	26	9.888	0.911 ± 0.077	0.00368 ± 0.00060
JB-ALA	10	7	29	29	9.888	0.911 ± 0.077	0.00368 ± 0.00035
JB-ULU	10	8	35	35	10.155	0.956 ± 0.059	0.00378 ± 0.00046
Overall	60	24	56	56	8.980	0.927 ± 0.020	0.00334 ± 0.00021

N, number of sequences; Nh, number of haplotypes; *S*, number of polymorphic sites; *m*, number of mutations; *k*, average number of nucleotide differences; *Hd*, haplotype diversity; *π*, nucleotide diversity; st. d., standard deviation.

**TABLE 2:** Uncorrected p-distances of Kazakhstani populations of *Equus caballus*.

Population	MZHR-NKZ	MZHR-KRG	KTN	AD	JB-ALA	JB-ULU
MZHR-NKZ	​	0.00064	0.00044	0.00075	0.00076	0.00070
MZHR-KRG	0.00305	​	0.00065	0.00078	0.00076	0.00074
KTN	0.00184	0.00287	​	0.00072	0.00072	0.00068
AD	0.00331	0.00393	0.00322	​	0.00074	0.00074
JB-ALA	0.00369	0.00388	0.00336	0.00389	​	0.00067
JB-ULU	0.00342	0.00392	0.00330	0.00370	0.00371	​

#### 2.3.2 Phylogenetic analysis

We retrieved from GenBank the sequences of *COI* and *cytb* regions from 151 previously published complete mitochondrial genomes and augmented them with our data set to evaluate the phylogenetic relationships of Kazakhstani horses with ancient and present-day horses (Lippold et al., 2011; Fages et al., 2019; Librado et al., 2015; Orlando et al., 2013; Schubert et al., 2014; Kusliy et al., 2021; Kalbfleisch et al., 2018; Xiao et al., 2016; Schubert et al., 2017) (Supplementary Table S1). As an outgroup we added the extinct equine species of North America, *Haringtonhippus francisci* (KT168317) (Heintzman et al., 2017; Vilstrup et al., 2013). The complete dataset for phylogenetic analysis comprised 211 sequences. Further estimation of phylogenetic relationships among haplotypes was performed using Bayesian Inference (BI) in MrBayes 3.2.6 (Ronquist et al., 2012). The best-fit codon-partitioning schemes and the best-fit substitution models (GTR) were selected using a version of jModelTest 2.1.10 (Darriba et al., 2012). according to the Bayesian information criterion (BIC). Phylogenetic analysis was performed by running Metropolis Markov chain Monte Carlo sampling with four chains for 10 million generations, and trees were sampled every 1000 generations. The first 20% of trees were discarded as burn-in after inspection for stationarity of log-likelihood scores in Tracer 1.7.1 (Rambaut et al., 2018). All parameters had an effective sample size [ESS] of >200. A majority-rule consensus tree was drawn from the post-burn-in samples, and posterior probabilities were calculated as the frequency of samples recovering any clade. Nodes with posterior probability/bootstrap values ≥ 0.95/≥75 were considered moderately or well supported. The relationships among haplotypes from all populations were inferred with NETWORK 4.6.0.0 (fluxus-engineering, 2026) using the median-joining method (Bandelt et al., 1999). The phylogenetic position of the studied haplotypes followed the default settings (r = 2, ε = 0) of network reconstruction (Jansen et al., 2002).

Further assessment of an internal phylogenetic relationships of Kazakhstani horses was executed *via* Neighbor-joining method in MEGA v. X (Kumar et al., 2018).

#### 2.3.3 Divergence time estimation

Divergence times among *Equus* lineages were estimated using a Bayesian relaxed molecular clock model with an uncorrelated lognormal distribution, as implemented in BEAST v.1.7 (Drummond et al., 2012). To improve model convergence and avoid over-parameterization, the analysis was performed using a reduced dataset focusing on caballine horses. We selected one or two representative samples from each breed or population included in this study, together with sequences retrieved from GenBank (Supplementary Table S2) (Achilli et al., 2012; Lippold et al., 2011; Librado et al., 2015; Orlando et al., 2013; Heintzman et al., 2017; Xu et al., 2007; Jiang et al., 2011; Der Sarkissian et al., 2015; Guo et al., 2016; Vorobieva et al., 2020; Kusliy et al., 2016; Yang et al., 2017; Sheikh et al., 2019; Vilstrup et al., 2013; Jonsson et al., 2014; Ibiş, 2019; Luo et al., 2011; Yuan et al., 2020). A total dataset (112 sequences) was included: (caballine horses: all available *E. caballus* breeds from GenBank representing global diversity and *E. przewalskii*); (extinct Pleistocene horses: *E. lambei*, *E. cf. scotti*, *E. dalianensis*) and (non-caballine *Equus* (crown group): zebras (*E. zebra*, *E. quagga*, *E. grevyi*), Asiatic asses (*E. hemionus*, *E. kiang*), African wild asses (*E. africanus*, *E. asinus*)). Outgroups were: *H. francisci* and *H. saldiasi*. The analysis was conducted using fossil-based calibration points: C1 (1.8 Mya) - *E. caballus*+*E. przewalskii* and C2 (5.6 Mya) - non-caballine *Equus* (Orlando et al., 2013; Vilstrup et al., 2013; Weinstock et al., 2005; Eisenman et al., 1992). Calibration priors were specified as lognormal distributions to account for uncertainty in node ages. Analyses were conducted under a Yule (pure-birth) (Ritchie et al., 2017) and birth-death speciation process to adequately determine the divergence time of Kazakhstani horses, along with other modern caballines. The best-fit model of nucleotide substitution for the aligned sequences was determined using jModelTest 2.1.10. The Markov chain Monte Carlo (MCMC) analysis was run for 50 million generations, with trees sampled every 1,000 generations. Convergence and effective sample sizes were assessed using Tracer. A maximum clade credibility tree was generated using TreeAnnotator, with node heights summarized as median values and a burn-in of 10%. The resulting tree was visualized using FigTree (FigTree v1.4.4, 2026).

#### 2.3.4 Population structure, differentiation, and demographic dynamics

To estimate genetic variation within populations, among populations within groups, and between groups, AMOVA was carried out in the program Arlequin v3.5 (Excoffier and Lischer, 2010), with significance tests based on 1000 permutations (Table 3). To identify groups of geographically homogeneous and genetically differentiated populations of Kazakhstani horse breeds, spatial analysis of molecular variance (SAMOVA) was performed using SAMOVA v2.0 (Dupanloup et al., 2002). The analysis iteratively partitions sampling locations into K groups (K = 2–5) by maximizing the proportion of total genetic variance attributable to differences among groups (FCT). For each value of K, 100 independent simulated annealing processes were conducted to ensure convergence. The optimal number of groups was determined based on the highest and/or plateauing FCT values, while avoiding configurations that produced single-population groups.

**TABLE 3:** Average node times derived from the BEAST based on Birth-death and Yule speciation process models.

Clade	Birth-death speciation Node age (95% HPD)	Yule speciation Node age (95% HPD)
C1 - 1.8 Mya (caballine horses, i.e., *E. przewalskii*), C2 - 5.6 Mya (non-caballine *Equus*)		
Non-caballine *Equus* (zebras, African wild asses, Asiatic asses) from MRCA	9.9 (5.05–18) Mya	5.03 (4.33–6.06) Mya
Within non-caballine *Equus*	5.6 (4.46–7.36) Mya	3.16 (1.76–4.62) Mya
NWSLH (*H. francisci*, *H. saldiasi*) from MRCA	—	3.25 (2.18–4.56) Mya
Within NWSLH	5.3 (1.49–11.11) Mya	2.05 (0.61–3.16) Mya
Pleistocene horses (*E. cf. Scotti*, *E. lambei*, *E. dalianensis*) from MRCA	2.53 (1.74–4.03)	2.38 (1.77–3.25) Mya
Within Pleistocene horses	1.22 (0.38–2.31) Mya	1.27 (0.41–2.17) Mya
*E. caballus Arab, CN, MNG*	1.75 (1.58–2.05) Mya	1.9 (1.6–2.42) Mya
*E. caballus* (common)	0.89 (0.48–1.53) Mya	1.73 (1.3–2.25) Mya

Genetic differentiation among populations was assessed using Arlequin v3.5 (Excoffier and Lischer, 2010). Pairwise F_ST_ values were calculated based on molecular distances derived from concatenated mtDNA sequence data using the pairwise differences method. The significance of F_ST_ values was evaluated using 10,000 permutations. As mitochondrial DNA reflects maternal inheritance, F_ST_ values were interpreted as indicators of historical patterns of maternal gene exchange among populations, with lower values suggesting higher levels of genetic proximity.

To determine the population dynamics and demographic patterns of the *E*. *caballus* populations from Kazakhstan, neutrality tests of Tajima`s *D* (Tajima, 1989), Fu`s *Fs* (Fu, 1997) and Ramos-Onsins R_2_ (Ramos-Onsins and Rozas, 2002) statistics were applied. Mismatch distribution analyses were conducted to examine past population expansion using the Harpending raggedness test (Rag) (Harpending, 1994) and the sum of squared deviations (SSD) (Table 4). The significance of the R_2_ and SSD statistics was assessed using coalescent simulations under a neutral model implemented in Arlequin v3.5 (Excoffier and Lischer, 2010). An approximate time since expansion was estimated from the τ parameter using the formula t = τ/(2u), where u represents the mutation rate per sequence per generation. Mutation rates were derived from BEAST analyses to ensure consistency with the phylogenetic framework.

**TABLE 4:** AMOVA analysis indices of Kazakhstani horses.

Source of variation	Sum of squares	Variance components	% variation
Among breeds	20.917	−0.15769	−3.48442
Among populations Within breeds	18.500	0.50741	11.21184
Among populations	225.500	4.17593	92.27258
Total	264.917	4.52564	​

## 3 Results

### 3.1 Genetic diversity

*COI* gene nucleotide frequencies were composed of 27.98% (A), 28.43% (T/U), 27.30% (C), and 16.30% (G). Among 1545 sites, 1517 were invariable (monomorphic) sites, and 28 variable (polymorphic) sites, including 9 singleton variable sites and 19 parsimony-informative sites. The total number of haplotypes is 18, with haplotype diversity (*Hd*) = 0.875 ± 0.030, and nucleotide diversity (*π*) = 0.00305 ± 0.00023.

For the *cytb* gene, nucleotide frequencies were 28.12% (A), 26.45% (T/U), 32.22% (C), and 13.20% (G), whereas among 1140 sites, 1112 were invariable and 28 were variable, including 8 singleton variable and 20 parsimony-informative sites. The total number of haplotypes is 17 with (*Hd*) = 0.897 ± 0.025, (*π*) = 0.00374 ± 0.00030 respectively.

The length of the concatenated dataset was 2687 bp. We recovered 99 haplotypes within 211 sequences from the dataset of ancient and modern horses, from which 24 haplotypes were obtained from 60 sequences of Kazakhstan horse populations, with the overall high haplotype diversity (*Hd*) = 0.927 ± 0.020, and nucleotide diversity (*π*) = 0.00334 ± 0.00021 respectively (Table 1).

Uncorrected pairwise differences between populations of Kazakhstani horse breeds are presented below the diagonal, standard error estimates are shown above the diagonal and were obtained by a bootstrap procedure (1000 replicates) following the Jukes-Cantor model. The genetic distance between two populations of Mugalzhar breed yielded a similarly close relationship of 0.06%, while to Adai and Jabe breed populations yielded 0.07% respectively, evidencing low genetic variability among the geographically and genetically proximate populations (Table 2).

### 3.2 Phylogenetic analysis

**FIGURE 2:**
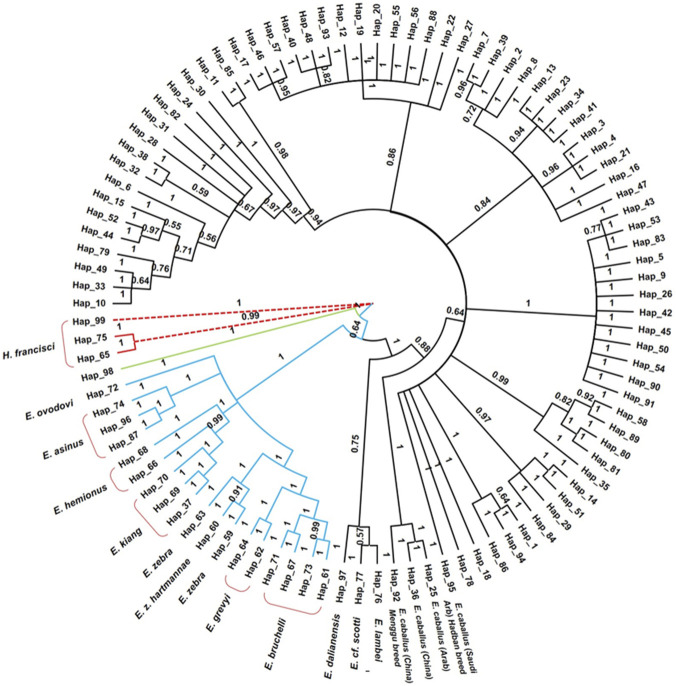
Phylogenetic relationships of Kazakhstani horses with other domestic and wild representatives of Equidae. Bootstrap supports are shown on the branches, respectively.

Among the 24 evolutionary models tested, the GTR model was identified as the best-fitting substitution model for all three partitions. The selected scheme yielded a log-likelihood (lnL) of −6204.3471 and a Bayesian Information Criterion (BIC) score of 13 722.8941. The resulting BI-tree exhibits localized polytomy within the caballine radiation, reflecting the close genetic relationships among modern domestic horses, breeds from Kazakhstan, and *E. przewalskii* (Figure 2). Most internal nodes show high statistical support (posterior probability ≥0.75). The only comparatively weakly supported node (posterior probability = 0.64) occurs at a basal position within the crown *Equus*, representing the relationship among major Equids, including the caballine clade relative to other extant groups.

Domestic horse haplotypes from Kazakhstan are distributed within the global caballine cluster rather than forming a geographically isolated lineage. These samples are interspersed among multiple modern domestic horse lineages worldwide and also occur in close phylogenetic proximity to *E. przewalskii* haplotypes, indicating shared maternal ancestry and a detectable signal of gene flow among lineages.

An internal genetic relationship assessed by the NJ-tree demonstrated no clear breed-specific clustering, further supporting the conclusion of weak genetic differentiation and a shared evolutionary history among the analyzed horse populations (Supplementary Figure S1). Due to the limited variability of the *COI* and *cytb* regions, haplogroup assignment was not attempted.

Marker-specific haplotype networks revealed the genetic relationships among Kazakhstani horse breeds, reflecting the impact of cross-breeding programs aimed at enhancing traits such as stamina, strong legs, or meat/milk precocity.

**FIGURE 3:**
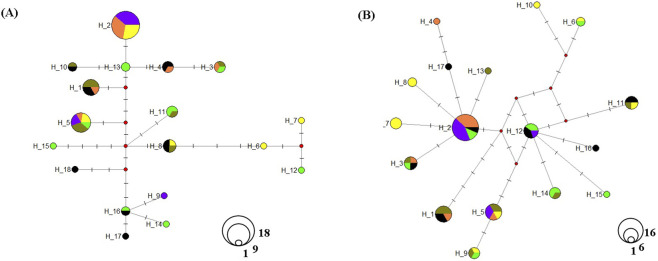
Median-joining haplotype network of *Equus caballus* populations from Kazakhstan. **(A)** COI gene haplotype network; **(B)** Cytb gene haplotype network. Colors are attributed as follows: MZHR-NKZ, orange; MZHR-KRG, black; AD, yellow; KTN, violet; JB-ALA, olive; JB-ULU, light green.

The *COI*-based network was characterized by a dominant haplotype (H_2, n = 18), which was shared among the KTN, AD, and MZHR-NKZ populations, indicating shared maternal ancestry or recent gene flow. Private haplotypes restricted to single breeds were also observed, including H_9 (KTN), H_6, 7 (AD), H_17, 18 (MZHR-KRG), and H_12, 14, 15 (JB-ULU) (Figure 3). In comparison, the *cytb*-based network exhibited a more pronounced star-like topology. While H_2 remained the most frequent haplotype (including individuals from MZHR-NKZ, MZHR-KRG, KTN, and JB-ULU), it was surrounded by several unique haplotypes representing breed-specific populations, such as Hap_10 (AD), Hap_4 (MZHR-NKZ), Hap_16, 17 (MZHR-KRG), Hap_13 (JB-ALA), and Hap_15 (JB-ULU) respectively (Figure 3).

### 3.3 Diversification time of Kazakhstani horses

**FIGURE 4:**
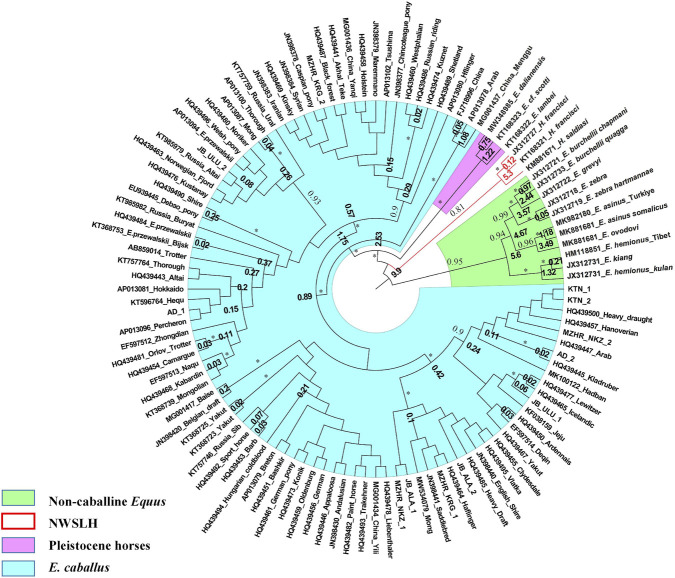
Divergence time from the MRCA of the Kazakhstani *Equus caballus* and other modern horse breeds under the birth-death evolutionary model. Node age (median heights) are given in bold. Posterior probabilities (PP) given in regular font, where * represents absolute support (100%), and those less than 50% are not shown.

We employed a relaxed (lognormal-uncorrelated) molecular clock, as implemented in BEAST v.1.7 (Drummond et al., 2012) to estimate the divergence time of Kazakhstani horses together with other domestic horses, *E. caballus* from the MRCA (Figure 4). We compared two different evolutionary speciation process models (birth-death and Yule). The resulting tree topologies were identical, and the obtained node ages from the MRCA are summarized in Table 3. NWSLH *- H. francisci* and *H. saldiasi* were chosen as an outgroup, but remain unconstrained.

Caballine and non-caballine horses represent monophyletic clades, in which the non-caballine *Equus* representatives diverged from the MRCA around 9.9 Mya (Miocene) following the birth-death (BD) speciation process and at 5.03 Mya (Pliocene) under pure-birth Yule (Y) speciation, while within-clade radiation occurred in the Pliocene at 5.6 Mya and 3.16 Mya respectively (Table 3). A further split occurred in New-World stilt-legged horses at 3.25 Mya and in extinct Pleistocene horses at 2.53 Mya (BD) and 2.38 Mya (Y).

### 3.4 Population-genetic structure and dynamics

Analysis of molecular variance (AMOVA) revealed that the majority of genetic variation was attributed to differences among populations (92.27%), whereas variation among breeds was negligible and negative (−3.48%) (Table 3). A smaller proportion of variation was observed among populations within breeds (11.21%) (Table 4).

The fixation indices further supported this pattern. The overall genetic differentiation among populations within breeds was moderate and statistically significant (FSC = 0.108, *p* < 0.001), as was the total genetic differentiation among populations (F_ST_ = 0.077, *p* < 0.001). In contrast, differentiation among breeds was not significant and slightly negative (FCT = −0.034, *p* = 0.883), indicating an absence of genetic structuring at the breed level.

SAMOVA identified the optimal grouping at K = 2 under the Kimura 2-parameter evolutionary model, in which group 1 represented by MZHR-NKZ and KTN populations, and group 2 (AD, MZHR-KRG, JB-ALA, and JB-ULU) respectively. Among all the yielded values of FCT (0.108) was the highest, indicating a shallow genetic differentiation among groups and geographical proximity. The consistency of this population clustering further supports the presence of underlying spatial genetic structure, indicating that geographic factors play a significant role in shaping genetic differentiation among the studied populations.

Pairwise F_ST_ values indicated generally low to moderate genetic differentiation among populations, with several comparisons showing non-significant differentiation. Negative and near-zero FST values were observed between some population pairs, suggesting a lack of genetic structure and extensive haplotype sharing. Significant differentiation (*p* < 0.05) was detected primarily between populations 1 (MZHR-NKZ) and populations 4–6 (JB-ALA, JB-ULU, MZHR-KRG), as well as between population 3 (KTN) and populations 4–6, with F_ST_ values ranging from 0.130 to 0.230 (Table 5). In contrast, populations 4 (JB-ALA), 5 (JB-ULU), and 6 (MZHR-KRG) exhibited low and non-significant F_ST_ values, indicating high genetic connectivity. Population 2 (AD) exhibited low and non-significant F_ST_ values with all other populations, indicating a lack of genetic differentiation and suggesting extensive genetic connectivity. Overall, these results support a pattern of low genetic differentiation and suggest substantial historical maternal gene flow among most populations.

**TABLE 5:** Population comparison differentiation of Kazakhstani horse breeds.

Population	MZHR-NKZ	AD	KTN	JB-ALA	JB-ULU	MZHR-KRG
MZHR-NKZ	—	​	​	​	​	​
AD	0.085	—	​	​	​	​
KTN	−0.023	0.024	—	​	​	​
JB-ALA	0.158*	0.056	0.130*	—	​	​
JB-ULU	0.230*	0.033	0.152*	0.034	—	​
MZHR-KRG	0.173*	0.044	0.140*	−0.012	−0.024	—

Statistical significance at p < 0.05 indicated by asterisks.

Neutrality test statistics indicated that Kazakhstani horse populations are generally consistent with mutation-drift equilibrium, with evidence of recent demographic expansion in several populations (Table 6). Tajima’s *D* values were predominantly negative but not significant in most populations, including Kazakh, Kostanay, and Mugalzhar (MZHR-NKZ), indicating no significant departure from neutrality. In contrast, the MZHR-KRG and AD populations exhibited positive Tajima’s *D* values; however, these were also not significant, providing no support for population contraction or balancing selection.

**TABLE 6:** Ramos-Onsins R2, Tajima`s *D* and Fu`s *Fs* neutrality test statistics (statistical significance *p* > 0.10). Mismatch distribution analysis (under the spatial expansion model) indices of populations of Kazakhstani *Equus caballus* inferred from concatenated *COI* and *cytb* gene sequences.

Population	*D*	*Fs*	R_2_	Rag (*p*-value)	SSD (*p*-value)	θ	τ
MZH-NKZ	−1.53910 *p* = 0.046	−5.91610 *p* = 0.000	0.1190	0.3027 *p* = 0.570	0.081 *p* = 0.430	3.606	1.394
MZHR-KRG	0.28390 *p* = 0.654	−3.44999 *p* = 0.035	0.1693	0.0840 *p* = 0.560	0.0234 *p* = 0.460	2.998	7.113
KTN	−0.20468 *p* = 0.434	−5.86104 *p* = 0.004	0.1761	0.5402 *p* = 0.490	0.110 *p* = 0.270	4.587	0.479
AD	0.36240 *p* = 0.710	−3.51441 *p* = 0.034	0.1704	0.1590 *p* = 0.400	0.0566 *p* = 0.280	4.808	5.080
JB-ALA	−0.16941 *p* = 0.465	−3.51441 *p* = 0.019	0.1357	0.0227 *p* = 0.970	0.0110 *p* = 0.900	2.290	6.899
JB-ULU	−0.86562 *p* = 0.231	−3.43737 *p* = 0.029	0.1163	0.1037 *p* = 0.290	0.038 *p* = 0.280	2.598	7.557
Overall	−0.85562 *p* = 0.217	−24.72027 *p* = 0.000	0.0767	0.0188 *p* = 0.780	0.0126 *p* = 0.470	3.748	5.233

R2 - Ramos-Onsins and Rozas statistics; Rag - Harpending raggedness statistics; SSD - sum of squared deviations; θ - estimates of Theta initial; τ - estimates of Tau.

Fu’s *Fs* statistics were largely negative and statistically significant across the majority of populations, indicating an excess of rare haplotypes and supporting a signal of recent population expansion. In contrast, weaker or less consistent values observed in MZHR-KRG and JB-ULU suggest heterogeneity in demographic patterns among populations.

The Ramos-Onsins and Rozas R_2_ statistic further supported these findings, with generally low values across populations and an overall R_2_ of 0.0767, consistent with recent demographic expansion at the species level. Nevertheless, variation in R_2_ values among populations indicates that the strength of this signal differs, with some populations showing patterns more consistent with demographic stability or only weak expansion.

**FIGURE 5:**
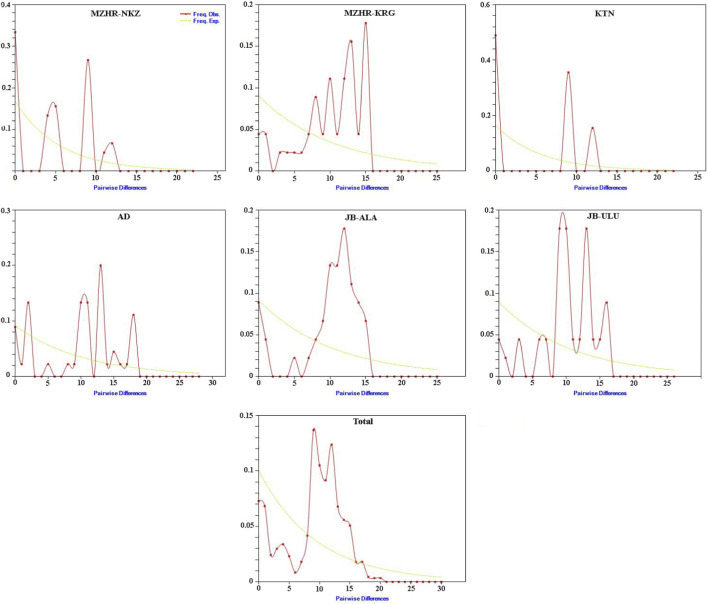
Mismatch distribution plots of Kazakhstani horse populations.

Mismatch distribution analyses conducted under the spatial expansion model revealed low and non-significant Harpending’s raggedness index (Rag) and SSD values across all populations, indicating no significant deviation from the expected expansion model. However, the observed mismatch distributions (Figure 5) were predominantly multimodal and irregular rather than smooth and unimodal. This pattern suggests that, despite statistical support for expansion, the demographic history of these populations is not consistent with a simple sudden expansion model.

Instead, the multimodal distributions observed in several populations (MZHR-KRG, AD, and JB-ULU) likely reflect population substructure, admixture among maternal lineages, or multiple demographic events, rather than a single expansion process. The combined evidence therefore indicates that Kazakhstani horse populations exhibit signals of expansion superimposed on complex and heterogeneous demographic histories.

An overall expansion time was estimated for the complete dataset to infer the general demographic history, whereas separate estimates at the breed level were not considered informative due to the lack of significant genetic differentiation among breeds. Mismatch distribution analysis of the concatenated mtDNA dataset (2685 bp) yielded a τ value of 5.233, which, in combination with the BEAST-derived mean substitution rate (6.3145 × 10^−3^ substitutions per site per million years) and an assumed generation time of 4 years, corresponds to an estimated population expansion time of approximately 205,864 years ago during the Late Pleistocene. The estimate reflects the overall demographic history of the dataset and is consistent with a scenario of population expansion likely influenced by Pleistocene climatic fluctuations, although its precise timing remains sensitive to assumptions regarding generation time.

## 4 Discussion

### 4.1 Genetic diversity and population structure

The present study reveals high haplotype diversity coupled with low nucleotide diversity in Kazakhstani populations of *E*. *caballus*, a pattern commonly observed in domestic species with complex breeding histories. Such a combination typically reflects the presence of multiple closely related maternal lineages that have accumulated only limited sequence divergence over time. This is consistent with the relatively low number of polymorphic sites detected across both the *COI* and *cytb* markers, as well as the low overall nucleotide diversity (π ≈ 0.0033). Comparatively, a similarly low level of nucleotide diversity (π = 0.0048) based on the cytb gene was observed in Kazakh horse populations from north-west China and adjacent Kazakhstan (Gemingguli et al., 2016). The high haplotype diversity (*Hd* = 0.927) based on a concatenated dataset was congruent with previous studies on Kazakh horses (Gemingguli et al., 2016) which yielded (0.989) and Kushum breed (0.922) based on the D-loop region of mtDNA (Nguyen et al., 2020). MtDNA analysis of Mongolian horses also revealed high diversity indices among populations (*π* = 0.021; *Hd* = 0.976) respectively (Bolor-Oyut et al., 2018).

Despite the high number of haplotypes identified (24 within Kazakhstan; 99 globally), genetic differentiation among populations is remarkably low, as evidenced by minimal pairwise distances (0.06%–0.07%) and low but significant F_ST_ values. The absence of significant differentiation among breeds (negative FCT) further indicates that breed designation does not reflect underlying mitochondrial genetic structure. Instead, most genetic variation is distributed within populations rather than among them.

Interestingly, very shallow genetic diversity was captured between Kazakh horses from Kazakhstan and Kazakh horses in China (>0.01%) (Gemingguli et al., 2016). It can be explained by the historical migration of the Kazakh population to adjacent north-west China during the collectivization period in Kazakhstan in 1930s. Horses, along with sheep, were brought to China by people, and they continued the breeding and pasturing system, which has conserved their genetic origin for nearly a century.

This pattern is likely driven by extensive gene flow and historical admixture, which is expected given the long-standing tradition of horse exchange, migration, and selective breeding across the Eurasian steppe. The weak population structure observed here aligns with previous findings in domestic horses, where maternal lineages are often widely shared across geographically distant populations.

### 4.2 Phylogenetic relationships and maternal lineage exchange

Phylogenetic reconstruction based on concatenated mitochondrial markers demonstrates that Kazakhstani horses are embedded within the broader caballine radiation rather than forming distinct or geographically isolated clades. The observed polytomy within the Bayesian tree reflects the shallow divergence and rapid radiation of domestic horse lineages, which has been widely reported in equine phylogenetic studies (Cieslak et al., 2010; Lippold et al., 2011; Orlando et al., 2013). In our study, the crown caballine horses were represented two main clades where Kazakhstani horses were scattered randomly along with other breeds such as Chinese Yanqi, Akhal-Teke, Russia riding, Syrian, Iranian, Russian Ural, Mongol, etc. With BS ranging from 0.84 to 1.0 respectively. Similar findings were captured in the study on Arabian horses (Almarzook et al., 2017) which demonstrated low distance values and no clear clustering in a phylogenetic position with other worldwide domestic horses. This suggests that Arabian horse strains show no genetic separation and share their mitochondrial haplotypes with breeds such as Akhal-Teke, Maremmano, Westphalian, Suffolk, Caspian pony, Deqin, Naqu, Iranian, etc.

The interspersed distribution of Kazakhstani haplotypes among global domestic horse lineages suggests multiple maternal origins and extensive historical connectivity. Moreover, it is important to emphasize the origin of the current Kazakhstani horse breed, which was established on the basis of the Kazakh horse type Jabe. Originally, Kazakh horses were somewhat close to the ancient ancestors of the Eurasian steppes and used to be a progenitor of all current Kazakhstani horse breeds, along with other world breeds such as the Orel Trotter, the Don breed, Akhal-Teke, Karabair, etc. (Kabylbekova et al., 2024), aiming to enhance physiological features such as productivity or resilience to year-long pastures in harsh environmental conditions in the Northern regions of the Central Asian Steppes.

The close phylogenetic proximity to *E*. *przewalskii* further supports the notion of shared ancestral polymorphism and/or historical introgression, consistent with previous genomic studies indicating gene flow between domestic and wild horse lineages (McCue et al., 2012).

Haplotype network analyses reinforce this interpretation. The presence of dominant, widely shared haplotypes (H_2_) across multiple populations indicates either recent common ancestry or ongoing gene flow. At the same time, the occurrence of private haplotypes within individual populations suggests localized diversification following admixture events.

The star-like topology observed in the *cytb* network is particularly indicative of population expansion, whereas the more structured *COI* network may reflect marker-specific differences in mutation rates or lineage sorting. Together, these results highlight a reticulate evolutionary history shaped by both shared ancestry and population-specific differentiation.

### 4.3 Population history dynamics

Neutrality tests and mismatch distribution analyses provide evidence for recent demographic expansion in Kazakhstani horse populations, although the signal is not uniform across all populations. The predominance of negative Fu’s *Fs* values and low R_2_ statistics suggests an excess of rare haplotypes, consistent with expansion following a bottleneck or founder event. However, the non-significant Tajima’s *D* values and the multimodal mismatch distributions indicate that the demographic history is more complex than a simple sudden expansion model. Instead, the observed patterns likely reflect multiple expansion events, population substructure, and admixture among maternal lineages.

Mismatch distribution analyses showed no significant deviation from the spatial expansion model, as indicated by low raggedness index and SSD values. However, the predominantly multimodal and irregular shape of the distributions suggests that the demographic history of Kazakhstani horse populations does not reflect a simple, single expansion event. Instead, these patterns are more consistent with population substructure, admixture among divergent maternal lineages, and/or multiple expansion episodes.

Estimates of expansion time further support this interpretation by indicating heterogeneous timing of demographic events across populations rather than a single synchronous expansion. These results suggest that population growth and dispersal occurred in multiple phases, likely associated with Late Pleistocene climatic fluctuations and ecological transitions, and were only later intensified by human-mediated movement following domestication. The inferred expansion time of ancient *E. caballus* populations, estimated at approximately 205,864 years ago, likely reflects a pre-domestication demographic expansion and is broadly consistent with the divergence estimate (∼0.29 Mya) obtained from the timetree analysis (Figure 4). Overall, while deep evolutionary splits within *Equus* shaped the ancient phylogenetic structure of the lineage, subsequent episodes of population expansion and admixture likely contributed to the high haplotypic diversity and weak phylogeographic structure observed in present-day Kazakhstani horses.

Although SAMOVA identified an optimal grouping at K = 2 (FCT = 0.108), indicating moderate genetic structuring, the AMOVA results revealed no significant differentiation among breeds (−3.4%) and between populations within breeds (11.2%), while the majority of variation occurred among populations (92.2%). This lack of clear phylogenetic separation among breeds suggests that breed designations do not correspond to distinct evolutionary lineages. Similar findings were reported by Gemingguli et al. (2018) (Gemingguli et al., 2016) on Kazakh horses from Kazakhstan and NW China, based on combined mtDNA markers, where the negative value of the variance was obtained among breeds (−0.91%), among populations within groups (5.35%), and within populations (95.56%). This fact suggests that population-level genetic variation appears to be structured primarily by geography rather than by breed-specific impact. Together, these findings indicate that the studied horse populations share a relatively recent common demographic history, with weak genetic structuring and substantial genetic variation retained within populations, supporting the interpretation of a broadly shared Late Pleistocene expansion event.

### 4.4 Implications for the evolutionary history of Kazakhstani horses

Taken together, our results support a scenario in which Kazakhstani horses exhibit shallow genetic differentiation despite high haplotypic diversity, reflecting a complex evolutionary history shaped by recurrent maternal gene flow and admixture, as well as long-term movements of horse populations across Eurasia. The lack of clear phylogeographic structure, together with extensive phylogenetic intermixing observed in the haplotype network and timetree (Figures 3, 4), indicates historically high connectivity among populations. These patterns are broadly consistent with sustained opportunities for gene flow over time in Central Asia, a region characterized by extensive human mobility and interregional contacts throughout the Holocene.

Rather than allowing direct attribution to specific historical processes, the genetic data more generally suggest repeated episodes of connectivity and admixture between local Kazakhstani horses and populations from adjacent regions, including present-day Russia, China, Mongolia, the Middle East, and Eastern Europe. While large-scale historical phenomena such as transcontinental exchange networks (the Silk Road), warfare, and nomadic pastoralism provide a plausible historical context for such connectivity, the mitochondrial data alone do not permit direct inference of their specific contributions or timing.

At the same time, the presence of private haplotypes and moderate within-population differentiation suggests that local diversification processes have persisted despite ongoing gene flow. This highlights a dual pattern in which regional evolutionary continuity coexists with intermittent external genetic inputs, ultimately generating a mosaic-like genetic structure.

From an evolutionary and archaeological perspective, the Eurasian steppes, particularly the territory of modern Kazakhstan, have long been proposed as a major center of horse domestication. Archaeological sites such as Botai and Borly, dated to approximately 5,000 years before present, provide some of the earliest evidence of horse management, rather than the onset of horse domestication. Recent paleogenomic studies demonstrated that the majority of present-day domestic horses derive from the DOM2 lineage that emerged in the Pontic–Caspian/Volga–Don steppes and rapidly expanded across Eurasia during the Late Bronze Age, largely replacing pre-existing Eurasian horse populations (Lira Garrido et al., 2025; Librado et al., 2021). This finding suggests that Botai horses likely represent an earlier and distinct management trajectory rather than the direct ancestors of modern domestic horses.

However, despite this significance, our mitochondrial data provide limited support for a clear, localized domestication signal. Instead, the high haplotypic diversity combined with weak population structure observed here is more consistent with extensive post-domestication admixture and lineage replacement, which may have obscured the genetic footprint of an initial domestication center.

Our divergence time estimates, as illustrated in the timetree (Figure 4), provide further insight into the deeper evolutionary framework underlying these patterns. The crown caballine lineage forms a well-supported clade distinct from non-caballine *Equus* (e.g., *E. asinus*, *E. hemionus*) and New World stilt-legged horses (*H. francisci*, *H. saldiasi*), while also showing clear separation from extinct Pleistocene taxa such as *E. lambei*, *E. cf. Scotti*, and *E. dalianensis*. The divergence of these Pleistocene equids was dated to approximately 2.53 Mya (95% HPD: 1.74–4.03 Mya), suggesting that they represent early lineages related to the ancestry of modern caballine horses.

Within the caballine clade, our estimates indicate that East Asian lineages, including populations from China, Japan, and Mongolia, diverged from the most recent common ancestor (MRCA) around 1.75 Mya (95% HPD: 1.58–2.05 Mya). Subsequent diversification within crown caballines was dated to approximately 0.89 Mya (95% HPD: 0.48–1.53 Mya). These results are consistent with a Pleistocene radiation of *E. caballus*, followed by population expansion and structuring across Eurasia (Lippold et al., 2011; Librado et al., 2021). Recent discoveries of Middle Pleistocene caballoid horses, identified as *Equus mosbachensis* (Schöningen horse), suggest a divergence time of approximately 570 kya (Weingarten et al., 2025), which closely matches our estimate (∼0.57 Mya) for the diversification of modern caballine horses following the separation of East Asian lineages.

Importantly, the topology of the timetree highlights the deep evolutionary separation among major equid lineages while simultaneously illustrating the relatively shallow divergence among modern domestic horses. This pattern supports the hypothesis that recent demographic processes, rather than ancient lineage divergence, play the dominant role in shaping present-day genetic diversity in Kazakhstani horse populations.

These findings are consistent with paleogenomic evidence indicating that equid evolution was strongly influenced by recurrent intercontinental dispersal events. Previous studies have demonstrated that multiple migration waves occurred *via* the Bering Land Bridge, which connected North America and Eurasia during the Pleistocene (Weingarten et al., 2025). Two major dispersal events have been proposed: (i) an early wave around 2.6 Mya, introducing the ancestors of non-caballine equids into Eurasia and Africa, and (ii) a later wave around 900–800 kya, associated with the emergence and expansion of caballine horses. The persistence of several Pleistocene lineages within the broader evolutionary trajectory leading to modern horses further underscores the long-term continuity of the caballine lineage across deep timescales.

Overall, divergence times inferred from non-caballine equids, crown caballines, New World stilt-legged horses, and extinct Pleistocene taxa demonstrate that the genetic landscape of modern Kazakhstani horses is embedded within a deep and reticulate evolutionary history shaped by ancient divergence, repeated dispersal events, and more recent anthropogenic influences.

Finally, it is important to emphasize that mitochondrial DNA reflects only maternal lineage history and represents a single, non-recombining locus. While it provides valuable insights into evolutionary history and diversification, it cannot fully resolve genome-wide patterns of admixture and population structure. Future studies incorporating nuclear genomic data (SNPs or whole-genome sequencing), broader geographic sampling, and additional ancient DNA will be essential to more precisely reconstruct the domestication history and subsequent diversification of horses across Central Asia.

## 5 Conclusion

This study provides new insights into the maternal genetic diversity, population structure, and evolutionary history of Kazakhstani horses based on complete mitochondrial DNA sequences. The observed pattern of high haplotypic diversity and low nucleotide diversity, together with weak population structure and extensive haplotype sharing, indicates a history of substantial gene flow and admixture across populations.

Phylogenetic and network analyses demonstrate that Kazakhstani horses are not genetically distinct but form part of a broader, globally interconnected caballine lineage. Demographic analyses further suggest that population expansion occurred in a complex and non-uniform manner, likely involving multiple phases of growth and secondary contact rather than a single expansion event. Divergence time estimates place the diversification of maternal lineages within a deep evolutionary framework, highlighting the importance of Pleistocene processes in shaping present-day genetic variation. These findings suggest that the current genetic landscape of Kazakhstani horses reflects the combined effects of ancient maternal lineage diversification, demographic expansion, and ongoing gene flow across Eurasia. Future studies incorporating nuclear genomic data and broader geographic sampling will be essential to further resolve population structure and better understand the evolutionary and domestication history of *E. caballus*.

## Data Availability

All newly generated sequences from this study were deposited in GenBank under accession numbers PZ226764-PZ226823; PZ232657-PZ232716.

## Supplementary material

SUPPLEMENTARY FIGURE S1 Neighbor-joining phylogenetic tree of Kazakhstani horses.

SUPPLEMENTARY TABLE S1 Sequence information analyzed in the phylogenetic analysis.

SUPPLEMENTARY TABLE S2 Sequence information employed in molecular clock analyses.
