# Supplementary material for: Mitochondrial DNA reveals high maternal diversity within a weak breed structure in native Kazakhstani horses
Source: Front Genet. 2026 Jul 6;17:1874969. doi: 10.3389/fgene.2026.1874969 (PMC13387962; doi:10.3389/fgene.2026.1874969)

Table S1. Sequence information analyzed in the phylogenetic analysis

| **Accession No.** | **Sequence information** | **Hap** | **Taxon** | **Source** |
| --- | --- | --- | --- | --- |
| **Sequences of Kazakhstani horses used in this study** | | | | |
| PZ232710 COI, PZ226814 Cytb | MZHR-KRG4 | Hap 1 | *Equus caballus* | This study |
| PZ232707 COI, PZ226817 Cytb | MZHR-KRG1 | Hap 1 | *Equus caballus* | This study |
| PZ232687 COI, PZ226800 Cytb | JB-ALA7 | Hap 1 | *Equus caballus* | This study |
| PZ232688 COI, PZ226795 Cytb | JB-ALA2 | Hap 1 | *Equus caballus* | This study |
| PZ232693 COI, PZ226794 Cytb | JB-ALA1 | Hap 1 | *Equus caballus* | This study |
| PZ232657 COI, PZ226764 Cytb | MZHR-NKZ1 | Hap 1 | *Equus caballus* | This study |
| PZ232686 COI, PZ226793 Cytb | KTN10 | Hap 2 | *Equus caballus* | This study |
| PZ232685 COI, PZ226792 Cytb | KTN9 | Hap 2 | *Equus caballus* | This study |
| PZ232684 COI, PZ226791 Cytb | KTN8 | Hap 2 | *Equus caballus* | This study |
| PZ232681 COI, PZ226788 Cytb | KTN5 | Hap 2 | *Equus caballus* | This study |
| PZ232679 COI, PZ226786 Cytb | KTN3 | Hap 2 | *Equus caballus* | This study |
| PZ232678 COI, PZ226785 Cytb | KTN2 | Hap 2 | *Equus caballus* | This study |
| PZ232677 COI, PZ226784 Cytb | KTN1 | Hap 2 | *Equus caballus* | This study |
| PZ232664 COI, PZ226771 Cytb | MZHR-NKZ8 | Hap 2 | *Equus caballus* | This study |
| PZ232663 COI, PZ226770 Cytb | MZHR-NKZ7 | Hap 2 | *Equus caballus* | This study |
| PZ232661 COI, PZ226768 Cytb | MZHR-NKZ5 | Hap 2 | *Equus caballus* | This study |
| PZ232660 COI, PZ226767 Cytb | MZHR-NKZ4 | Hap 2 | *Equus caballus* | This study |
| PZ232659 COI, PZ226766 Cytb | MZHR-NKZ3 | Hap 2 | *Equus caballus* | This study |
| PZ232658 COI, PZ226765 Cytb | MZHR-NKZ2 | Hap 2 | *Equus caballus* | This study |
| PZ232662 COI, PZ226769 Cytb | MZHR-KRG6 | Hap 3 | *Equus caballus* | This study |
| PZ232697 COI, PZ226804 Cytb | JB-ULU1 | Hap 3 | *Equus caballus* | This study |
| PZ232694 COI, PZ226801 Cytb | JB-ALA8 | Hap 3 | *Equus caballus* | This study |
| PZ232662 COI, PZ226769 Cytb | MZHR-NKZ6 | Hap 3 | *Equus caballus* | This study |
| PZ232665 COI, PZ226772 Cytb | MZHR-NKZ9 | Hap 4 | *Equus caballus* | This study |
| PZ232691 COI, PZ226798 Cytb | JB-ALA5 | Hap 5 | *Equus caballus* | This study |
| PZ232690 COI, PZ226797 Cytb | JB-ALA4 | Hap 5 | *Equus caballus* | This study |
| PZ232682 COI, PZ226789 Cytb | KTN6 | Hap 5 | *Equus caballus* | This study |
| PZ232680 COI, PZ226787 Cytb | KTN4 | Hap 5 | *Equus caballus* | This study |
| PZ232669 COI, PZ226776 Cytb | AD3 | Hap 5 | *Equus caballus* | This study |
| PZ232666 COI, PZ226773 Cytb | MZHR-NKZ10 | Hap 5 | *Equus caballus* | This study |
| PZ232667 COI, PZ226774 Cytb | AD1 | Hap 6 | *Equus caballus* | This study |
| PZ232676 COI, PZ226783 Cytb | AD10 | Hap 7 | *Equus caballus* | This study |
| PZ232670 COI, PZ226777 Cytb | AD4 | Hap 7 | *Equus caballus* | This study |
| PZ232668 COI, PZ226775 Cytb | AD2 | Hap 7 | *Equus caballus* | This study |
| PZ232674 COI, PZ226781 Cytb | AD8 | Hap 8 | *Equus caballus* | This study |
| PZ232671 COI, PZ226778 Cytb | AD5 | Hap 8 | *Equus caballus* | This study |
| PZ232706 COI, PZ226813 Cytb | JB-ULU10 | Hap 9 | *Equus caballus* | This study |
| PZ232696 COI, PZ226803 Cytb | JB-ALA10 | Hap 9 | *Equus caballus* | This study |
| PZ232672 COI, PZ226779 Cytb | AD6 | Hap 9 | *Equus caballus* | This study |
| PZ232673 COI, PZ226780 Cytb | AD7 | Hap 10 | *Equus caballus* | This study |
| PZ232715 COI, PZ226822 Cytb | MZHR-KRG9 | Hap 11 | *Equus caballus* | This study |
| PZ232711 COI, PZ226818 Cytb | MZHR-KRG5 | Hap 11 | *Equus caballus* | This study |
| PZ232695 COI, PZ226802 Cytb | JB-ALA9 | Hap 11 | *Equus caballus* | This study |
| PZ232675 COI, PZ226782 Cytb | AD9 | Hap 11 | *Equus caballus* | This study |
| PZ232683 COI, PZ226790 Cytb | KTN7 | Hap 12 | *Equus caballus* | This study |
| PZ232689 COI, PZ226796 Cytb | JB-ALA3 | Hap 13 | *Equus caballus* | This study |
| PZ232692 COI, PZ226799 Cytb | JB-ALA6 | Hap 14 | *Equus caballus* | This study |
| PZ232701 COI, PZ226808 Cytb | JB-ULU5 | Hap 14 | *Equus caballus* | This study |
| PZ232698 COI, PZ226805 Cytb | JB-ULU2 | Hap 14 | *Equus caballus* | This study |
| PZ232699 COI, PZ226806 Cytb | JB-ULU3 | Hap 15 | *Equus caballus* | This study |
| PZ232703 COI, PZ226810 Cytb | JB-ULU7 | Hap 16 | *Equus caballus* | This study |
| PZ232700 COI, PZ226807 Cytb | JB-ULU4 | Hap 16 | *Equus caballus* | This study |
| PZ232702 COI, PZ226809 Cytb | JB-ULU6 | Hap 17 | *Equus caballus* | This study |
| PZ232704 COI, PZ226811 Cytb | JB-ULU8 | Hap 18 | *Equus caballus* | This study |
| PZ232705 COI, PZ226812 Cytb | JB-ULU9 | Hap 19 | *Equus caballus* | This study |
| PZ232708 COI, PZ226815 Cytb | MZHR-KRG2 | Hap 19 | *Equus caballus* | This study |
| PZ232709 COI, PZ226816 Cytb | MZHR-KRG3 | Hap 20 | *Equus caballus* | This study |
| PZ232713 COI, PZ226820 Cytb | MZHR-KRG7 | Hap 21 | *Equus caballus* | This study |
| PZ232714 COI, PZ226821 Cytb | MZHR-KRG8 | Hap 22 | *Equus caballus* | This study |
| PZ232716 COI, PZ226823 Cytb | MZHR-KRG10 | Hap 23 | *Equus caballus* | This study |
| **Global Caballine Horses** | | | | |
| MW534079 |  | Hap 1 |  | Kusliy et al., 2021 [35] |
| KT757762 |  | Hap 1 |  | Orlando et al., 2013 [33] |
| JN398441 |  | Hap 1 |  | Achilli et al., 2012 [14] |
| JN398440 |  | Hap 1 |  | Achilli et al., 2012 [14] |
| HQ439495 |  | Hap 1 |  | Lippold et al., 2011 [30] |
| HQ439485 |  | Hap 1 |  | Lippold et al., 2011 [30] |
| HQ439466 |  | Hap 1 |  | Lippold et al., 2011 [30] |
| HQ439464 |  | Hap 1 |  | Lippold et al., 2011 [30] |
| HQ439455 |  | Hap 1 |  | Lippold et al., 2011 [30] |
| HQ439442 |  | Hap 1 |  | Lippold et al., 2011 [30] |
| HQ439500 |  | Hap 2 |  | Lippold et al., 2011 [30] |
| HQ439457 |  | Hap 2 |  | Lippold et al., 2011 [30] |
| HQ439447 |  | Hap 2 |  | Lippold et al., 2011 [30] |
| HQ439444 |  | Hap 2 |  | Lippold et al., 2011 [30] |
| MG001434 |  | Hap 5 |  | Yang et al., 2017 [53] |
| MG001424 |  | Hap 5 |  | Yang et al., 2017 [53] |
| JN398430 |  | Hap 5 |  | Achilli et al., 2012 [14] |
| HQ439493 |  | Hap 5 |  | Lippold et al., 2011 [30] |
| HQ439482 |  | Hap 5 |  | Lippold et al., 2011 [30] |
| HQ439473 |  | Hap 5 |  | Lippold et al., 2011 [30] |
| HQ439470 |  | Hap 5 |  | Lippold et al., 2011 [30] |
| HQ439461 |  | Hap 5 |  | Lippold et al., 2011 [30] |
| HQ439456 |  | Hap 5 |  | Lippold et al., 2011 [30] |
| HQ439446 |  | Hap 5 |  | Lippold et al., 2011 [30] |
| KT368724 |  | Hap 11 |  | Librado et al., 2015 [32] |
| KT985979 |  | Hap 14 |  | Vorobieva et al., 2020 [51] |
| JN398416 |  | Hap 14 |  | Achilli et al., 2012 [14] |
| HQ439496 |  | Hap 14 |  | Lippold et al., 2011 [30] |
| HQ439476 |  | Hap 14 |  | Lippold et al., 2011 [30] |
| HQ439463 |  | Hap 14 |  | Lippold et al., 2011 [30] |
| AP013094 |  | Hap 14 |  | Wada et al., 2014 unpubl. |
| AP013088 |  | Hap 14 |  | Wada et al., 2014 unpubl. |
| KF038159 |  | Hap 16 |  | Yoon S.H., 2014 unpubl. |
| JN398391 |  | Hap 17 |  | Achilli et al., 2012 [14] |
| MG001440 |  | Hap 18 |  | Yang et al., 2017 [53] |
| HQ439490 |  | Hap 18 |  | Lippold et al., 2011 [30] |
| MG001436 |  | Hap 19 |  | Yang et al., 2017 [53] |
| MG001432 |  | Hap 19 |  | Yang et al., 2017 [53] |
| KT368757 |  | Hap 19 |  | Der Sarkissian et al., 2015 [49] |
| KT368738 |  | Hap 19 |  | Librado et al., 2015 [32] |
| JN398386 |  | Hap 19 |  | Achilli et al., 2012 [14] |
| JN398385 |  | Hap 19 |  | Achilli et al., 2012 [14] |
| JN398384 |  | Hap 19 |  | Achilli et al., 2012 [14] |
| JN398383 |  | Hap 19 |  | Achilli et al., 2012 [14] |
| JN398378 |  | Hap 19 |  | Achilli et al., 2012 [14] |
| HQ439488 |  | Hap 19 |  | Lippold et al., 2011 [30] |
| HQ439475 |  | Hap 19 |  | Lippold et al., 2011 [30] |
| HQ439469 |  | Hap 19 |  | Lippold et al., 2011 [30] |
| HQ439458 |  | Hap 19 |  | Lippold et al., 2011 [30] |
| HQ439441 |  | Hap 19 |  | Lippold et al., 2011 [30] |
| AP013102 |  | Hap 19 |  | Wada et al., 2014 unpubl. |
| AP013101 |  | Hap 19 |  | Wada et al., 2014 unpubl. |
| HQ439477 |  | Hap 21 |  | Lippold et al., 2011 [30] |
| HQ439465 |  | Hap 21 |  | Lippold et al., 2011 [30] |
| HQ439448 |  | Hap 21 |  | Lippold et al., 2011 [30] |
| HQ439489 |  | Hap 22 |  | Lippold et al., 2011 [30] |
| AB859014 | Trotter breed | Hap 24 | *Equus caballus* | Wada et al., 2014 unpubl. |
| AP013078 | AR1, Arab breed | Hap 25 | *Equus caballus* | Wada et al., 2014 unpubl. |
| AP013079 | BR1, Breton breed | Hap 26 | *Equus caballus* | Wada et al., 2014 unpubl. |
| AP013080 | HF1, Hflinger breed | Hap 27 | *Equus caballus* | Wada et al., 2014 unpubl. |
| AP013081 | Hk1, Hokkaido native breed, Japan | Hap 28 | *Equus caballus* | Wada et al., 2014 unpubl. |
| AP013100 | TH1, Thoroughbred breed | Hap 29 | *Equus caballus* | Wada et al., 2014 unpubl. |
| AP013087 | MO1, Mongolian native breed | Hap 29 | *Equus caballus* | Wada et al., 2014 unpubl. |
| KT368754 | Ewld2_Bijsk2, historical | Hap 30 | *Equus przewalski* | Der Sarkissian et al., 2015 [49] |
| KT368753 | Ewld1_Bijsk1, historical | Hap 30 | *Equus przewalskii* | Der Sarkissian et al., 2015 [49] |
| HQ439484 |  | Hap 30 | *Equus przewalskii* | Lippold et al., 2011 [30] |
| AP013095 |  | Hap 30 | *Equus przewalskii* | Wada et al., 2014 unpubl. |
| MH586816 | Twilight, Thoroughbred breed | Hap 31 | *Equus caballus* | Kalbfleisch et al., 2018 [36] |
| KT757764 | Twiilight, Thoroughbred breed | Hap 31 | *Equus caballus* | Orlando et al., 2013 [33] |
| AP013096 | PR1, Percheron breed, Japan | Hap 31 | *Equus caballus* | Wada et al., 2014 unpubl. |
| EF597512 | Zhongdian breed, China | Hap 32 | *Equus caballus* | Xu et al., 2007 [47] |
| KT368758 | Ewld5_Theodor, historical, Hybrid | Hap 33 | *Equus przewalskii* | Der Sarkissian et al., 2015 [49] |
| KT368739 | Emgl1, Mongolian breed, historical | Hap 33 | *Equus caballus* | Der Sarkissian et al., 2015 [49] |
| HQ439468 | Kabardin breed | Hap 33 | *Equus caballus* | Lippold et al., 2011 [30] |
| EF597513 | Naqu breed, China | Hap 33 | *Equus caballus* | Xu et al., 2007 [47] |
| EF597514 | Deqin breed, China | Hap 34 | *Equus caballus* | Xu et al., 2007 [47] |
| EU939445 | Debao breed, China | Hap 35 | *Equus caballus* | Jiang et al., 2011 [48] |
| FJ718996 | TN8947, China | Hap 36 | *Equus caballus* | Ning et al., 2016 unpubl. |
| HQ439443 | Altai breed | Hap 38 | *Equus caballus* | Lippold et al., 2011 [30] |
| HQ439445 | Kladruber breed | Hap 39 | *Equus caballus* | Lippold et al., 2011 [30] |
| MG001426 | Chakouyi, HPG C, China | Hap 40 | *Equus caballus* | Yang et al., 2017 [53] |
| HQ439487 | Black Forest | Hap 40 | *Equus caballus* | Lippold et al., 2011 [30] |
| HQ439449 | Syr2, Arab breed | Hap 40 | *Equus caballus* | Lippold et al., 2011 [30] |
| HQ439450 | Ardennais breed | Hap 41 | *Equus caballus* | Lippold et al., 2011 [30] |
| HQ439451 | Bashkir Curly breed | Hap 42 | *Equus caballus* | Lippold et al., 2011 [30] |
| HQ439453 | Barb breed | Hap 43 | *Equus caballus* | Lippold et al., 2011 [30] |
| HQ439452 | Bashkir Curly breed | Hap 43 | *Equus caballus* | Lippold et al., 2011 [30] |
| HQ439454 | Camargue breed | Hap 44 | *Equus caballus* | Lippold et al., 2011 [30] |
| HQ439459 | Oldenburg breed | Hap 45 | *Equus caballus* | Lippold et al., 2011 [30] |
| KT368727 | Horse_1, Yakutia | Hap 46 | *Equus caballus* | Librado et al., 2015 [32] |
| JN398389 | 13_Syr05, Syria | Hap 46 | *Equus caballus* | Achilli et al., 2012 [14] |
| HQ439486 | Russian riding breed | Hap 46 | *Equus caballus* | Lippold et al., 2011 [30] |
| HQ439474 | Kuznet breed | Hap 46 | *Equus caballus* | Lippold et al., 2011 [30] |
| HQ439460 | Westphalian breed | Hap 46 | *Equus caballus* | Lippold et al., 2011 [30] |
| HQ439467 | Yakut breed | Hap 47 | *Equus caballus* | Lippold et al., 2011 [30] |
| KT368726 | CGG101397, Ancient horse, Yakutia | Hap 48 | *Equus caballus* | Librado et al., 2015 [32] |
| HQ439471 | Kladruber breed | Hap 48 | *Equus caballus* | Lippold et al., 2011 [30] |
| HQ439472 | Kladruber breed | Hap 49 | *Equus caballus* | Lippold et al., 2011 [30] |
| HQ439478 | Wild1, Liebenthaler breed | Hap 50 | *Equus caballus* | Lippold et al., 2011 [30] |
| HQ439480 | Noriker breed | Hap 51 | *Equus caballus* | Lippold et al., 2011 [30] |
| HQ439481 | Orlov Trotter breed | Hap 52 | *Equus caballus* | Lippold et al., 2011 [30] |
| HQ439492 | Spotted horse breed | Hap 53 | *Equus caballus* | Lippold et al., 2011 [30] |
| HQ439494 | Hungarian Coldblood breed | Hap 54 | *Equus caballus* | Lippold et al., 2011 [30] |
| JN398377 | 1_ChP01, Chincoteague pony breed, North America | Hap 55 | *Equus caballus* | Achilli et al., 2012 [14] |
| JN398379 | 3_Mrm12_Italy | Hap 56 | *Equus caballus* | Achilli et al., 2012 [14] |
| JN398390 | 14_Ita01_Italy | Hap 57 | *Equus caballus* | Achilli et al., 2012 [14] |
| JN398420 | 45_Bel01 | Hap 58 | *Equus caballus* | Achilli et al., 2012 [14] |
| KT368725 | Ancient horse, Yakutia, Batagai, Russia | Hap 78 | *Equus caballus* | Librado et al., 2015 [32] |
| KT368723 | Ancient horse, Yakutia, Yukagir, Russia | Hap 78 | *Equus caballus* | Librado et al., 2015 [32] |
| KT368741 | Mongolian horse, KB7754 | Hap 79 | *Equus caballus* | Der Sarkissian et al., 2015 [49] |
| KT368755 | Historical holotype | Hap 80 | *Equus przewalskii* | Der Sarkissian et al., 2015 [49] |
| KT368756 | paratype | Hap 81 | *Equus przewalskii* | Der Sarkissian et al., 2015 [49] |
| KT596764 | Hequ breed, China | Hap 82 | *Equus caballus* | Guo et al., 2015 [50] |
| KT757746 | JW25/MS299, Siberia, Russia | Hap 83 | *Equus caballus* | Orlando et al., 2013 [33] |
| KT757759 | JW374/MS303, Ural, Russia | Hap 84 | *Equus caballus* | Orlando et al., 2013 [33] |
| KT985982 | ZAR19, Buryatia, Russia | Hap 85 | *Equus caballus* | Kusliy et al., 2016 [52] |
| KT998647 | Jianchang breed, China | Hap 86 | *Equus caballus* | Wang et al., 2016 [37] |
| MG001416 | Debao, HPG A, China | Hap 88 | *Equus caballus* | Yang et al., 2017 [53] |
| MG001417 | Baise, HPG J, China | Hap 89 | *Equus caballus* | Yang et al., 2017 [53] |
| MG001425 | Tengchong 2, HPG L, China | Hap 90 | *Equus caballus* | Yang et al., 2017 [53] |
| MG001433 | Jinjiang, HPG L, China | Hap 91 | *Equus caballus* | Yang et al., 2017 [53] |
| MG001437 | Menggu, HPG R, China | Hap 92 | *Equus caballus* | Yang et al., 2017 [53] |
| MG001438 | Lijiang, HPG C, China | Hap 93 | *Equus caballus* | Yang et al., 2017 [53] |
| MG001439 | Changdu HPG N, China | Hap 94 | *Equus caballus* | Yang et al., 2017 [53] |
| **Global Non-caballine Horses** | | | | |
| JX312718 | 1041 | Hap 59 | *Equus zebra* | Vilstrup et al., 2013 [55] |
| JX312719 | H21 | Hap 60 | *Equus zebra* | Vilstrup et al., 2013 [55] |
| JX312724 | H11 | Hap 63 | *Equus zebra* | Vilstrup et al., 2013 [55] |
| JX312722 | G51 | Hap 62 | *Equus grevyi* | Vilstrup et al., 2013 [55] |
| JX312723 | G42 | Hap 62 | *Equus grevyi* | Vilstrup et al., 2013 [55] |
| JX312725 | 6390 | Hap 64 | *Equus grevyi* | Vilstrup et al., 2013 [55] |
| HM118851 | Tibet, China | Hap 37 | *Equus hemionus* | Luo et al., 2011 [58] |
| JX312728 | Ehemionus | Hap 66 | *Equus hemionus kulan* | Vilstrup et al., 2013 [55] |
| JX312730 | O91 | Hap 68 | *Equus hemionus onager* | Vilstrup et al., 2013 [55] |
| JX312729 | CGG10086 | Hap 67 | *Equus burchellii chapmani* | Vilstrup et al., 2013 [55] |
| JX312733 | QUAGGA | Hap 71 | *Equus bruchellii quagga* | Vilstrup et al., 2013 [55] |
| KM881680 |  | Hap 73 | *Equus bruchellii quagga* | Jonsson et al., 2014 [56] |
| JX312721 | 6381 | Hap 61 | *Equus bruchellii* | Vilstrup et al., 2013 [55] |
| JX312731 | K41 | Hap 69 | *Equus kiang* | Vilstrup et al., 2013 [55] |
| JX312732 | K32 | Hap 70 | *Equus kiang* | Vilstrup et al., 2013 [55] |
| JX312734 | ACAD2304 | Hap 72 | *Equus ovodovi* | Vilstrup et al., 2013 [55] |
| KM881681 |  | Hap 74 | *Equus asinus somalicus* | Jonsson et al., 2014 [56] |
| KX669267 |  | Hap 87 | *Equus asinus africanus* | Shubert et al., 2017 [38] |
| MK982180 | Anatolia donkey | Hap 96 | *Equus asinus* | Ibis, 2019 [57] |
| **New-Worl Stilt-Legged Horses** | | | | |
| KT168321 | PH047, Yukon, Canada | Hap 75 | *Haringtonhippus francisci* | Heintzman et al., 2017 [39] |
| KT168317 | PH014 | Hap 99 | *Haringtonhippus francisci* | Heintzman et al., 2017 [39] |
| JX312727 | New world SL | Hap 65 | *Equus sp.(Haringtonhippus francisci)* | Vilstrup et al., 2013 [55] |
|  |  |  |  |  |
| KM881671 | AE5717 | Hap 98 | *Hippidion saldiasi* | Der Sarkissian et al., 2015 |
| **Extinct Pleistocene Horses** | | | | |
| KT168322 | MS316, Yukon, Canada | Hap 76 | *Equus lambei* | Heintzman et al., 2017 [39] |
| KT168323 | PH053, Yukon, Canada | Hap 77 | *Equus cf. scotti* | Heintzman et al., 2017 [39] |
| MW348985 | DLH2 | Hap 97 | *Equus dalianensis* | Yuan et al., 2020 [59] |

**Table S2.** Sequence information employed in molecular clock analyses

| **GenBank**  **Acc. №** | **Voucher** | **Locality** | **Species (breed)** | **Source** |
| --- | --- | --- | --- | --- |
| **Caballine horses** | | | | |
| PZ226764 COI/PZ232657 Cytb | MZHR_NKZ_1 | North Kazakhstan region, Kazakhstan | *E. caballus* Mugalzhar breed | This study |
| PZ226765 COI/PZ232658 Cytb | MZHR_NKZ_2 | North Kazakhstan region, Kazakhstan | *E. caballus* Mugalzhar breed | This study |
| PZ226814 COI/PZ232707 Cytb | MZHR_KRG_1 | Karaganda region, Kazakhstan | *E. caballus* Mugalzhar breed | This study |
| PZ226815 COI/PZ232708 Cytb | MZHR_KRG_2 | Karaganda region, Kazakhstan | *E. caballus* Mugalzhar breed | This study |
| PZ232667 COI/PZ226774 Cytb | AD_1 | Mangystau region, Kazakhstan | *E. caballus* Aday breed | This study |
| PZ232668 COI/PZ226775 Cytb | AD_2 | Mangystau region, Kazakhstan | *E. caballus* Aday breed | This study |
| PZ232677 COI/PZ226784 Cytb | KTN_1 | Kostanay region, Kazakhstan | *E. caballus* Kostanay breed | This study |
| PZ232678 COI/PZ226785 Cytb | KTN2 | Kostanay region, Kazakhstan | *E. caballus* Kostanay breed | This study |
| PZ232687 COI/PZ226794 Cytb | JB_ALA_1 | Almaty region, Kazakhstan | *E. caballus* Kazakh breed, Jabe type | This study |
| PZ232688 COI/PZ226795 Cytb | JB_ALA_2 | Almaty region, Kazakhstan | *E. caballus* Kazakh breed, Jabe type | This study |
| PZ232697 COI/PZ226804 Cytb | JB_ULU_1 | Ulytau region, Kazakhstan | *E. caballus* Kazakh breed, Jabe type | This study |
| PZ232697 COI/PZ226805 Cytb | JB_ULU_2 | Ulytau region, Kazakhstan | *E. caballus* Kazakh breed, Jabe type | This study |
| AB859014 |  | Japan, Hokkaido | *E. caballus* Trotter | Wada et al., 2014 unpubl. |
| AP013078 |  | Japan, Hokkaido | *E. caballus* Arab | Wada et al., 2014 unpubl. |
| AP013079 |  | Japan, Hokkaido | *E. caballus* Breton | Wada et al., 2014 unpubl. |
| AP013080 |  | Japan, Hokkaido | *E. caballus* Hflinger | Wada et al., 2014 unpubl. |
| AP013081 |  | Japan, Hokkaido | *E. caballus* Hokkaido native | Wada et al., 2014 unpubl. |
| AP013087 |  | Japan, Hokkaido | *E. caballus* Mongolian native | Wada et al., 2014 unpubl. |
| AP013094 |  | Japan, Hokkaido | *E. przewalskii* | Wada et al., 2014 unpubl. |
| AP013096 |  | Japan, Hokkaido | *E. caballus* Percheron | Wada et al., 2014 unpubl. |
| AP013100 |  | Japan, Hokkaido | *E. caballus* Thoroughbred | Wada et al., 2014 unpubl. |
| AP013102 |  | Japan, Hokkaido | *E. caballus* Tsushima native | Wada et al., 2014 unpubl. |
| EF597512 |  | China | *E. caballus* Zhongdian | Xu et al., 2007 [47] |
| EF597513 |  | China | *E. caballus* Naqu | Xu et al., 2007 [47] |
| EF597514 |  | China | *E. caballus* Deqin | Xu et al., 2007 [47] |
| EU939445 |  | China | *E. caballus* Debao pony | Jiang et al., 2011 [48] |
| FJ718996 |  | China | *E. caballus* | Ning et al., 2016 unpubl. |
| HQ439441 |  |  | *E. caballus* Akhal-Teke | Lippold et al., 2011 [30] |
| HQ439443 |  |  | *E. caballus* Altai | Lippold et al., 2011 [30] |
| HQ439445 |  |  | *E. caballus* Kladruber | Lippold et al., 2011 [30] |
| HQ439446 |  |  | *E. caballus* Appaloosa | Lippold et al., 2011 [30] |
| HQ439447 |  |  | *E. caballus* Arab | Lippold et al., 2011 [30] |
| HQ439450 |  |  | *E. caballus* Ardennais | Lippold et al., 2011 [30] |
| HQ439451 |  |  | *E. caballus* Bashkir Curly | Lippold et al., 2011 [30] |
| HQ439453 |  |  | *E. caballus* Barb | Lippold et al., 2011 [30] |
| HQ439454 |  |  | *E. caballus* Camargue | Lippold et al., 2011 [30] |
| HQ439455 |  |  | *E. caballus* Clydesdale | Lippold et al., 2011 [30] |
| HQ439456 |  |  | *E. caballus* German sport horse | Lippold et al., 2011 [30] |
| HQ439457 |  |  | *E. caballus* Hanoverian | Lippold et al., 2011 [30] |
| HQ439458 |  |  | *E. caballus* Holstein | Lippold et al., 2011 [30] |
| HQ439459 |  |  | *E. caballus* Oldenburg | Lippold et al., 2011 [30] |
| HQ439460 |  | Central Europe | *E. caballus* Westphalian | Lippold et al., 2011 [30] |
| HQ439461 |  |  | *E. caballus* German riding pony | Lippold et al., 2011 [30] |
| HQ439463 |  |  | *E. caballus* Norwegian Fiord | Lippold et al., 2011 [30] |
| HQ439464 |  |  | *E. caballus* Haflinger | Lippold et al., 2011 [30] |
| HQ439465 |  |  | *E. caballus* Icelandic horse | Lippold et al., 2011 [30] |
| HQ439467 |  |  | *E. caballus* Yakut | Lippold et al., 2011 [30] |
| HQ439468 |  |  | *E. caballus* Kabardin | Lippold et al., 2011 [30] |
| HQ439469 |  |  | *E. caballus* Kinsky | Lippold et al., 2011 [30] |
| HQ439473 |  |  | *E. caballus* Konik | Lippold et al., 2011 [30] |
| HQ439474 |  |  | *E. caballus* Kuznet | Lippold et al., 2011 [30] |
| HQ439476 |  |  | *E. caballus* Kustanay | Lippold et al., 2011 [30] |
| HQ439477 |  |  | *E. caballus* Lewitzer | Lippold et al., 2011 [30] |
| HQ439478 |  |  | *E. caballus* Liebenthaler | Lippold et al., 2011 [30] |
| HQ439480 |  |  | *E. caballus* Noriker | Lippold et al., 2011 [30] |
| HQ439481 |  |  | *E. caballus* Orlov Trotter | Lippold et al., 2011 [30] |
| HQ439482 |  |  | *E. caballus* Paint horse | Lippold et al., 2011 [30] |
| HQ439484 |  |  | *E. przewalskii* | Lippold et al., 2011 [30] |
| HQ439485 |  |  | *E. caballus* Rhineland Heavy Draft | Lippold et al., 2011 [30] |
| HQ439486 |  |  | *E. caballus* Russian riding horse | Lippold et al., 2011 [30] |
| HQ439487 |  |  | *E. caballus* Black forest | Lippold et al., 2011 [30] |
| HQ439489 |  |  | *E. caballus* Shetland | Lippold et al., 2011 [30] |
| HQ439490 |  |  | *E. caballus* Shire | Lippold et al., 2011 [30] |
| HQ439492 |  |  | *E. caballus* Sport horse | Lippold et al., 2011 [30] |
| HQ439493 |  |  | *E. caballus* Trakehner | Lippold et al., 2011 [30] |
| HQ439494 |  |  | *E. caballus* Hungarian coldblood | Lippold et al., 2011 [30] |
| HQ439495 |  |  | *E. caballus* Vlatka | Lippold et al., 2011 [30] |
| HQ439496 |  |  | *E. caballus* Welsh pony | Lippold et al., 2011 [30] |
| HQ439500 |  |  | *E. caballus* Vladimir heavy draught | Lippold et al., 2011 [30] |
| JN398377 |  | North America | *E. caballus* Chincoteague Pony | Achilli et al., 2012 [14] |
| JN398378 |  | Middle East | *E. caballus* Caspian pony | Achilli et al., 2012 [14] |
| JN398379 |  | Italy | *E. caballus* Maremmano | Achilli et al., 2012 [14] |
| JN398383 |  | Iran | *E. caballus* | Achilli et al., 2012 [14] |
| JN398384 |  | Syria | *E. caballus* | Achilli et al., 2012 [14] |
| JN398420 |  | Belgium | *E. caballus* Belgian draft | Achilli et al., 2012 [14] |
| JN398430 |  | Spain | *E. caballus* Andalusian | Achilli et al., 2012 [14] |
| JN398440 |  | United Kingdom | *E. caballus* English Shire | Achilli et al., 2012 [14] |
| JN398441 |  | North America | *E. caballus* Saddlebred | Achilli et al., 2012 [14] |
| KT368723 |  | Russia, Yakutia | *E. caballus* Ancient Yakut Horse Yukagir | Librado et al., 2015 [32] |
| KT368725 |  | Russia, Yakutia | *E. caballus* Ancient Yakut Horse Batagai | Librado et al., 2015 [32] |
| KT368739 |  | Mongolia | *E. caballus* Mongolian Historical Horse | Der Sarkissian et al., 2015 [49] |
| KT368753 |  |  | *E. przewalskii* Bijsk Historical Horse | Der Sarkissian et al., 2015 [49] |
| KT596764 |  | China | *E. caballus* Hequ | Guo et al., 2015 [50] |
| KT757746 |  | Russia, Siberia | *E. caballus* | Orlando et al., 2013 [33] |
| KT757759 |  | Russia, Ural | *E. caballus* | Orlando et al., 2013 [33] |
| KT757764 |  |  | *E. caballus* Twilight Thoroughbred | Orlando et al., 2013 [33] |
| KT985979 |  | Russia, Altai, Ukok | *E. caballus* | Vorobieva et al., 2020 [51] |
| KT985982 |  | Russia, Buryatia | *E. caballus* | Kusliy et al., 2016 [52] |
| MW534079 |  | Mongolia | *E. caballus* | Kusliy et al., 2021 [35] |
| MG001417 |  | China | *E. caballus* Baise | Yang et al., 2017 [53] |
| MG001434 |  | China | *E. caballus* Yili | Yang et al., 2017 [53] |
| MG001436 |  | China | *E. caballus* Yanqi | Yang et al., 2017 [53] |
| MG001437 |  | China | *E. caballus* Menggu | Yang et al., 2017 [53] |
| MK100122 |  | Saudi Arabia | *E. caballus* Hadban | Sheikh et al., 2019 [54] |
| **Non-caballine horses** | | | | |
| JX312718 |  |  | *E. zebra* | Vilstrup et al., 2013 [55] |
| JX312719 |  |  | *E. zebra hartmannae* | Vilstrup et al., 2013 [55] |
| JX312721 |  |  | *E. burchellii chapmani* | Vilstrup et al., 2013 [55] |
| JX312722 |  |  | *E. grevyi* | Vilstrup et al., 2013 [55] |
| JX312728 |  |  | *E. hemionus kulan* | Vilstrup et al., 2013 [55] |
| JX312731 |  |  | *E. kiang* | Vilstrup et al., 2013 [55] |
| JX312733 |  |  | *E.burchellii_quagga* | Vilstrup et al., 2013 [55] |
| JX312734 |  |  | *E. ovodovi* | Vilstrup et al., 2013 [55] |
| KF038159 |  | Korea | *E. caballus* Jeju | Yoon, S.H., 2014 unpubl. |
| KM881681 |  |  | *E. asinus somalicus* | Jonsson et al., 2014 [56] |
| MK982180 |  | Turkey | *E. asinus* | Ibis, 2019 [57] |
| KT168322 |  | Canada, Yukon, Klondike | *E. lambei* | Heintzman et al., 2017 [39] |
| KT168323 |  | Canada, Yukon, Klondike | *E. cf. scotti* | Heintzman et al., 2017 [39] |
| HM118851 |  | China, Tibet | *E. hemionus* (Tibetan wild ass) | Luo et al., 2011 [58] |
| MW348985 |  | China | *E. dalianensis* | Yuan et al., 2020 [59] |
| KM881671 |  |  | *Hippodion saldiasi* | Der Sarkissian et al., 2015 [49] |
| JX312727 |  |  | *Haringtonhippus francisci* (NWSL) | Vilstrup et al., 2013 [55] |
| KT168321 |  | Canada, Yukon, Klondike | *Haringtonhippus francisci* | Heintzman et al., 2017 [39] |

References to Tables S1 - S2.

Note: citations given in the brackets [] are according to the reference list of the main text. Unpublished citations are given below, according to GenBank information.

Wada, K.; Yoshida, S.; Okubo, S.; Seki, Y.; Kikkawa, Y. and Yokohama, M. Whole mitochondrial genome analysis reveals that Hokkaido population of the Japanese native horse is composed of two divergent maternal lineages. 2014. Unpublished.

Ning, T.; Xiao, H.; Wang, G.-D.; Li, J. and Zhang, Y.-P. Selection tendency in Chinese domestic horse. 2016. Unpublished.

Yoon, S.H. Domestication History of the Horse: An Updated Genome-Wide Perspective Derived from Mitochondrial Genome Sequences. 2014. Unpublished.

Zhao, C.; Ma, H.; Xiang, H. The whole mtDNA of China native horse. 2017. Unpublished.

Figure S1. Neighbor-joining phylogenetic tree of Kazakhstani horses.


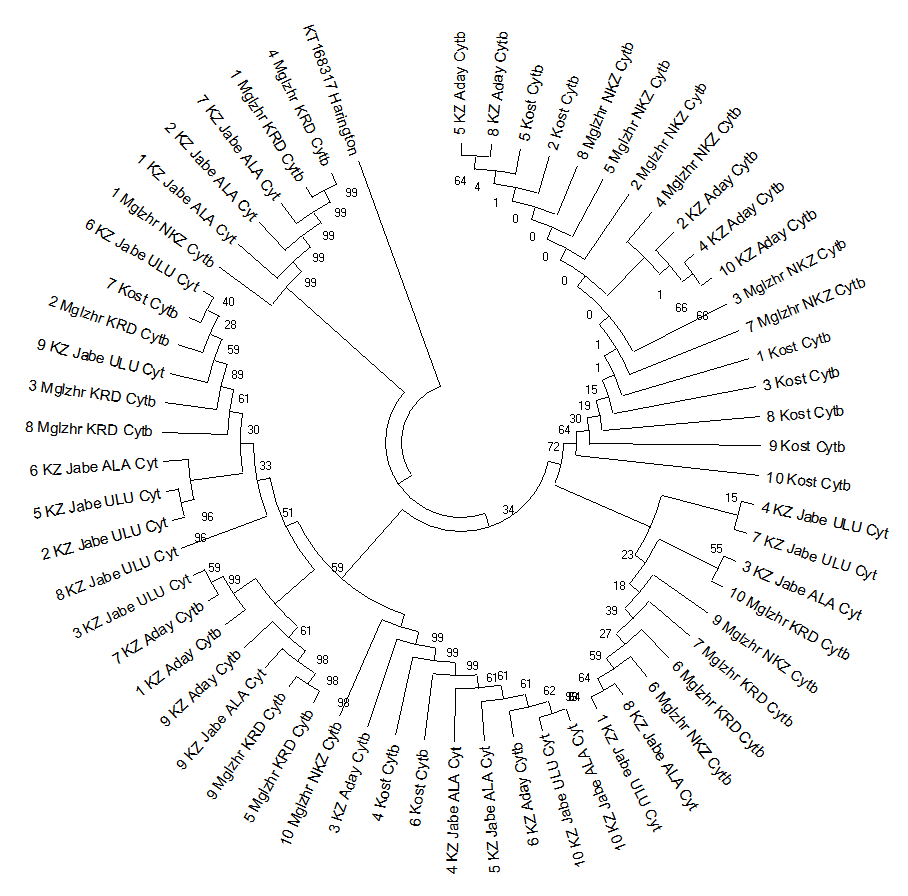

Supplement: Supplementary file 1 [file DataSheet1.docx]
